# Screening for sickle cell disease in newborns: a systematic review

**DOI:** 10.1186/s13643-020-01504-5

**Published:** 2020-10-30

**Authors:** Britta Runkel, Birgit Klüppelholz, Anne Rummer, Wiebke Sieben, Ulrike Lampert, Claudia Bollig, Martina Markes, Ulrike Paschen, Konstanze Angelescu

**Affiliations:** 1 Institute for Quality and Efficiency in Health Care, Cologne, Germany; 2 Institute for Evidence in Medicine, Medical Center - University of Freiburg, Faculty of Medicine, University of Freiburg, Freiburg, Germany; 3 Cochrane Germany, Cochrane Germany Foundation, Freiburg, Germany

**Keywords:** Newborn screening, Anaemia—sickle cell, SCD screening, Systematic review

## Abstract

**Background:**

Sickle cell disease (SCD) is an inherited autosomal recessive disorder caused by the replacement of normal haemoglobin (HbA) by mutant Hb (sickle Hb, HbS). The sickle-shaped red blood cells lead to haemolysis and vaso-occlusion. Especially in the first years of life, patients with SCD are at high risk of life-threatening complications. SCD prevalence shows large regional variations; the disease predominantly occurs in sub-Saharan Africa. We aimed to systematically assess the evidence on the benefit of newborn screening for SCD followed by an earlier treatment start.

**Methods:**

We systematically searched bibliographic databases (MEDLINE, EMBASE, Cochrane Databases, and the Health Technology Assessment Database), trial registries, and other sources to identify systematic reviews and randomised controlled trials (RCTs) or non-randomised trials on newborn screening for SCD. The last search was in 07/2020. Two reviewers independently reviewed abstracts and full-text articles and assessed the risk of bias of the studies included. Data were extracted by one person and checked by another. As meta-analyses were not possible, a qualitative summary of results was performed.

**Results:**

We identified 1 eligible study with direct evidence: a Jamaican retrospective study evaluating newborn screening for SCD followed by preventive measures (prevention of infections and education of parents). The study included 500 patients with SCD (intervention group, 395; historical control group, 105). Although the results showed a high risk of bias, the difference between the intervention and the control group was very large: mortality in children decreased by a factor of about 10 in the first 5 years of life (0.02% in the intervention group vs. 0.19% in the control group, odds ratio 0.09; 95% confidence interval [0.04; 0.22], *p* < 0.001).

**Conclusion:**

The results are based on a single retrospective study including historical controls. However, the decrease of mortality by a factor of 10 is unlikely to be explained by bias alone. Therefore, in terms of mortality, data from this single retrospective study included in our systematic review suggest a benefit of newborn screening for SCD (followed by preventive measures) versus no newborn screening for SCD (weak certainty of conclusions).

**Supplementary Information:**

The online version contains supplementary material available at 10.1186/s13643-020-01504-5.

## Background

Sickle cell disease (SCD) is an inherited autosomal recessive haemoglobin (Hb) disorder caused by the replacement of normal Hb (HbA) by mutant Hb (sickle Hb, HbS). In addition to homozygous SCD (SCD-S/S), compound heterozygous forms exist that lead to a variable clinical picture. The 3 most common genotypes of SCD are SCD-S/S, SCD-S/C, and SCD-S/beta^0^ thalassaemia [1].

SCD-S/S is the most common type of sickle cell disease; copies of the haemoglobin gene are inherited from both parents. SCD-S/C is the second most common type. The HbC gene is inherited from one parent and the HbS gene from the other. People with SCD-S/C have symptoms similar to those with SCD-S/S. SCD-S/beta^0^ thalassaemia is a combination of the sickle cell mutation and beta-thalassaemia; one parent passes on a sickle cell allele, the other a beta-thalassaemia allele. No HbA is produced.

Other rarer combinations are also known [2]. The production of abnormal sickle-shaped red blood cells causes haemolysis and vaso-occlusion [1]. Symptoms typically start in the first years of life. Common symptoms include pain, infections, and anaemic symptoms. Young children are particularly at high risk of life-threatening complications such as sepsis and acute splenic sequestration. The global prevalence of SCD is estimated at 2.28 per 1000 persons [3]. It varies greatly from region to region and correlates with the prevalence of malaria, as carriers of the HbS mutation are less susceptible to malaria. SCD predominantly occurs in sub-Saharan Africa, as well as in parts of the eastern Mediterranean, the Middle East and India, and has been spread globally by migration [2, 4]. The prevalence estimates per 1000 persons are 10.68 in Africa and 0.07 in Europe [3]. It is estimated that 230,000 children with SCD are born in sub-Saharan Africa per year (0.74% of births), compared with only 1300 children in Europe (< 0.1% of births) [2]. It can be assumed that in European countries, SCD occurs only in descendants from the regions mentioned above.

A US guideline on newborn screening [5], the British National Health Service [6], and the World Health Organization [7] all recommend screening for SCD to ensure that affected newborns receive the necessary care quickly and to reduce mortality and morbidity.

Management strategies aim to avoid the symptoms and complications of triggering factors and vaso-occlusive crises [8–12]. The German guideline of the Consortium of the German Society for Paediatric Oncology and Haematology [13] recommends preventive behavioural measures for SCD management (including awareness of signs of acute complications, see also [8]), prevention of infections (including penicillin prophylaxis and vaccinations, see also [9–12]), and lifelong, structured monitoring and treatment [13].

SCD can be diagnosed by a blood sample. Biochemical methods such as isoelectric focusing, capillary electrophoresis or high-performance liquid chromatography (HPLC) have previously been used for blood analysis; more recent methods include mass spectroscopy and molecular genetic analysis [2, 14].

The aim of newborn screening using a blood sample is the early detection and management of specific diseases. In the current German newborn screening programme [15], venous or heel blood is collected in the 36th to 72nd hour of life, dripped onto filter paper cards, and examined for several target diseases, which currently do not include SCD. In contrast, newborn screening for SCD has been established in the USA [5], England [16], France [17], Spain [18], Belgium [19], and the Netherlands [20].

This systematic review was conducted to support the decision on whether Germany should launch a national SCD screening programme. However, this work can also serve as a guidance for other health care systems that have not yet established such a programme. The aim of the current article was to systematically review the evidence on the benefit of newborn screening for SCD followed by an earlier start of treatment versus either no newborn screening for SCD or versus newborn screening for SCD (= diagnosis of SCD) not followed by any further measures. The focus of the assessment was on patient-relevant outcomes (mortality, morbidity [e.g. pain, infections, hospitalisations], adverse events, and health-related quality of life).

## Methods

### Protocol and methodological approach

The review formed part of a health technology assessment (HTA) conducted by the German Institute for Quality and Efficiency in Health Care (Institut für Qualitaet und Wirtschaftlichkeit im Gesundheitswesen, IQWiG). The full (German-language) report and protocol (Commission No. S18-01) are available on the Institute’s website (www.iqwig.de). IQWiG’s responsibilities and methodological approach are described in its methods paper [21]. This review was written according to the PRISMA statement [22] (see Additional file 1). Only previously published studies were used, so an ethical review and patient consent were not required.

### Eligibility criteria

The target population comprised newborns preferably investigated in controlled intervention studies of the screening and management strategy. The intervention was screening for SCD, with subsequent initiation of treatment. The timing of the intervention and diagnostic measures were to be comparable to those of the German newborn screening programme, which ranges from the 36th to the 72nd hour of life and uses blood samples taken from a vein or the heel dripped onto filter paper cards. The control intervention was no screening for SCD with subsequent initiation of treatment (either no screening for SCD or screening for SCD [= diagnosis of SCD] not followed by any further measures). The patient-relevant outcomes investigated included overall mortality of children with SCD, morbidity (e.g. pain, infections, and hospitalisations), any adverse events reported, and the health-related quality of life of the child (measured by any validated scale).

Randomised controlled trials (RCTs) were the primary type of evidence to be included in this review. If RCT data were insufficient, non-randomised comparative intervention studies and controlled cohort studies, including retrospective or historical comparisons (comparison of the later intervention group with an earlier, i.e. non-concurrent, control group), were to be considered.

If such studies with direct evidence were not available or were of insufficient quantity or quality, a linked evidence approach was to be used. Merlin et al. defined the full linked evidence approach as “the synthesis of systematically acquired evidence on the accuracy of a medical test, its impact on clinical decision making and the effectiveness of consequent treatment options” and presented several variations and abridged approaches [23]. Here, linked evidence from studies on diagnostic accuracy was to be used, together with controlled intervention studies investigating the benefit and harm of an earlier start of treatment for newborns with SCD. Due to the fact that direct evidence was identified and considered sufficient to draw conclusions (at least on the weakest level according to IQWiG’s grading system, see Table 1), the linked evidence approach is not further described here.

**Table 1:** Certainty of conclusions regularly inferred for different evidence situations if studies with the same qualitative certainty of results are available according to IQWiG methods

		Number of studies				
		1 (with statistically significant effect)	≥ 2			
			Homogenous	Heterogeneous		
			Meta-analysis statistically significant	Effects in the same direction^a^		
				Clear	Moderate	No
**Qualitative certainty of results**	High	Indication	Proof	Proof	Indication	-
	Moderate	Hint	Indication	Indication	Hint	-
	Low	-	Hint	Hint	-	-

^a^Effects in the same direction are present if a clear or moderate direction is recognizable despite of heterogeneity

There was no restriction to the duration of the studies, the length of follow-up, and settings.

### Search strategy and study selection

We searched for relevant primary studies and secondary publications (systematic reviews and HTA reports) in MEDLINE via PubMed and via Ovid (1946 to July 2020), EMBASE (1974 to July 2020), and the Cochrane Central Register of Controlled Trials (July 2020) as well as trial registries (ClinicalTrials.gov and EU Clinical Trials Register). The Cochrane Database of Systematic Reviews (Cochrane Reviews), MEDLINE, EMBASE, and the Health Technology Assessment Database (Technology Assessments) were searched for relevant systematic reviews. Reference lists of relevant systematic reviews were screened for additional relevant primary studies. There were no restrictions on language or date of publication. Conference abstracts were not eligible for inclusion. The search strategies, which were developed by one information specialist and checked by another, are presented in Additional file 2. After conducting the search, duplicates were removed using the reference management software Endnote X9 (by Clarivate, Philadelphia, USA). The remaining references were then screened with the in-house web trial selection database (webTSDB).

Two reviewers independently screened titles and abstracts of the citations retrieved to identify potentially eligible primary and secondary publications. The full texts of these articles were obtained and independently evaluated by the same two reviewers applying the full set of inclusion and exclusion criteria. Disagreements were resolved by consensus.

### Data extraction

The individual steps of the data extraction and risk-of-bias assessment procedures were always conducted by one person and checked by another; disagreements were resolved by consensus. Details of the studies were extracted using standardized tables developed and routinely used by IQWiG.

We extracted information on study characteristics from each study considered (including study design, sample size, place, and period in which the study was conducted, characteristics of the intervention group, characteristics of the control group, main inclusion and exclusion criteria, and patient-relevant outcomes).

### Assessment of risk of bias

We assessed the risk of bias for individual studies, as well as for each outcome, and rated these risks as “high” or “low”.

For controlled intervention studies, the risk of bias was assessed by determining the adequacy of the following quality criteria, which closely follow the criteria of the Cochrane risk-of-bias tool [24]: treatment groups were studied in parallel, comparability of groups, blinding of participants and investigators, selective outcome reporting, absence of other factors potentially causing bias, and overall risk of bias (study level). If the risk of bias on the study level was rated as “high”, the risk of bias on the outcome level was also generally rated as “high”. Study results were only considered in the analyses if at least 70% of the study participants had been evaluated.

### Data analysis

We reported the treatment effects as odds ratios (ORs) for binary outcomes, including 95% confidence intervals (CIs) and *p* values (Wald test).

Sensitivity analyses were planned in cases where the effect observed might not have been robustly estimated or unreliable due to methodological factors (e.g. algorithms for replacing missing values, choice of cut-offs for defining outcome variables or length of follow-up when outcomes were measured at several points in time). We planned subgroup analyses to examine the impact of variables such as sex, age, or type of diagnostic test on the effect of the intervention.

All calculations were performed with the statistical software R6.3.6 [25].

### Assessment of certainty of evidence

Using the IQWiG methods [21], we graded the results of the analysis into different categories with regard to the respective qualitative certainty of the conclusions (see Table 1). The data provide either “proof” (highest certainty of conclusions), an “indication” (medium certainty of conclusions), or a “hint” (weakest certainty of conclusions) in respect of the benefit or harm of an intervention—or none of these 3 categories apply.

## Results

### Literature search

**Fig. 1:**
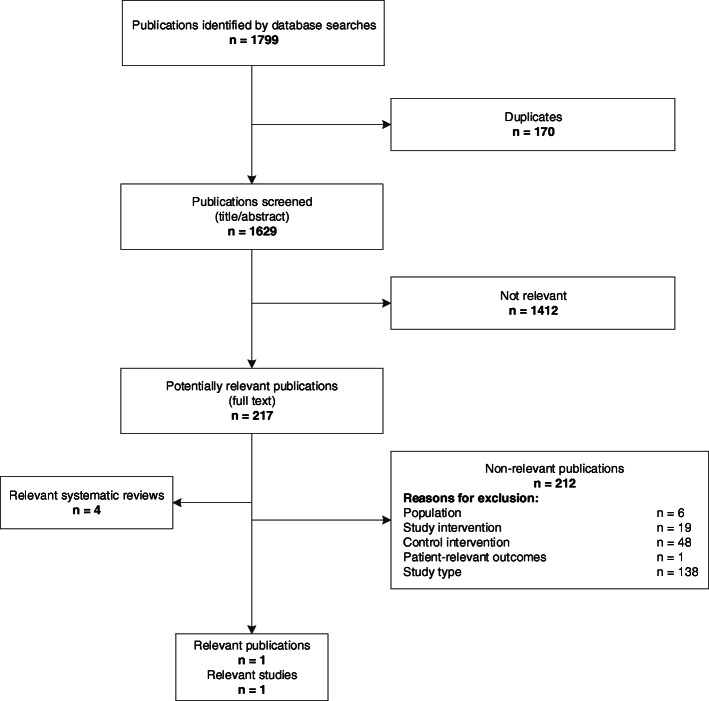
Flowchart of study selection for direct evidence

Only one controlled intervention study of the screening and management strategy was identified: King et al. [26]. The flowchart of study selection is presented in Fig. 1. Because of the direct evidence available in this study, we dispensed with a search for linked evidence. A list of excluded full texts with reasons for exclusion is presented in Additional file 3.

### Study characteristics

King et al. [26] was a retrospective study evaluating the screening programme for SCD in Jamaica. The intervention group included 395 SCD-S/S-positive infants who had been identified by screening (followed by preventive measures) between 1995 and 2006. Management of SCD in the intervention group included prevention of infections (e.g. penicillin prophylaxis) and education of parents (e.g. how to perform splenic palpation). The control group were participants in a historical cohort observation study initiated to provide information on the course of SCD, including 105 SCD-S/S-positive infants identified by screening between 1973 and 1975 (Jamaican Sickle Cell Cohort Study [27, 28]). These infants remained untreated, as no established treatment for SCD was available at that time. Further characteristics of the study are shown in Table 2.

**Table 2:** Study characteristics

Study	Study design	Investigated newborns (intervention/control)	Number of newborns with SCD	Place and period of recruitment	Intervention/control	Main inclusion criteria	Patient-relevant endpoints	Funding source
King et al. [26]	Retrospective controlled cohort study	*Intervention group*: 150,803	435^a^ (SCD-S/S)	Jamaica Victoria Jubilee Hospital, Kingston, 11/1995–07/2006 University Hospital of the West Indies, Kingston, 10/1997–07/2006 Spanish Town Hospital, St. Catherine, 04/1998–07/2006	- Screening of live newborns for SCD - In the case of diagnosis of an SCD initial consultation and education programme - If possible, parents will receive a newborn first consultation at the clinic before the 4th month of life - Guidance of the parents on how to perform a splenic palpation - From the 4th month of life penicillin prophylaxis - Every 3 months routine examination in clinic, every 6 months after 5 years of age	- Consecutive live newborns - Screening of umbilical cord blood indicates SCD-S/S phenotype - Confirmation diagnostics confirmed SCD-S/S (electrophoresis)	Mortality in 1st, 2nd, 3rd, 5th, and 10th year of life^b^	Not stated
		*Control group*^c^: approx. 30,000	105 (SCD-S/S)	Jamaica Victoria Jubilee Hospital, Kingston, 06/1973–12/1975	-Screening of all live newborns for SCD -Follow-up every 3 months			

*SCD-S/S* homozygous sickle cell disease

^a^Parents of 40 of the 435 newborns did not attend the initial consultation. Therefore, 395 newborns were included in the intervention programme

^b^Other reported patient-relevant endpoints (hospitalisations, “serious illness”, and invasive pneumococcal disease) were not considered, as their operationalisations were unusable

^c^First subpopulation of the birth cohort 06/1973–12/1981 (*N* = 100,000) of Victoria Jubilee Hospital, Kingston, Jamaica. The second (recruitment: 12/1975–01/1979) and third subpopulations (recruitment: 01/1979–12/1981) are not presented in the report because in these subpopulations the diagnosis of SCD was associated with secondary preventive measures; see Lee 1995 [25]

### Risk of bias

King et al.’s study [26] was rated as having a high risk of bias (Table 3). This was due to the historical control group and the lack of control for confounding factors. As the data were collected retrospectively, a lack of blinding was assumed. No baseline data on the newborns in the intervention and control group were available, but since they were born in the same region, comparability was assumed. The high risk of bias at the study level had a direct impact on the risk of bias at the outcome level. The risk of bias for the outcome “mortality” was thus also rated as high.

**Table 3:** Risk of bias in the study included (King et al. [26])

Study	Both treatment groups studied in parallel (yes/no)	Comparability of groups or adequate control for confounding factors	Blinding patient/investigator	Selective reporting improbable	Absence of other factors potentially causing bias	Risk of bias (study level)
King et al. [26]	No	Unclear^a^	No/no	Yes	Yes	High

^a^No control for potential confounding factors. No baseline data available. Comparability of groups in terms of baseline characteristics seems likely, as newborns of the same region were included in both groups

### Effects of newborn screening for SCD (Table 4)

Within the first year of life, 0.01% of newborns with SCD-S/S died in the intervention group, versus 0.10% in the control group. There was a clear difference between groups at 1 year with an OR of 0.09 (95% CI [0.03; 0.30]) and a *p* value of *p* < 0.001. This means that for the intervention group (cohort 1995–2006), the odds of dying were reduced to as little as 9% of the odds for the control group (cohort 1973–1975).

**Table 4:** Results on mortality

Study	Age	Intervention		Control		Intervention vs. control		
		***n***	Mortality rate^**a**^ (%) [95% CI]	***n***	Mortality rate^**a**^ (%) [95% CI]	OR^**b**^	[95% CI]^**b**^	***p*** value^**b**^
King et al. [26]		395^c^		105				
	1st year of life	n.a.	0.01 [0.01; 0.03]	n.a.	0.10 [0.04; 0.15]	0.09	[0.03; 0.30]	< 0.001
	2nd year of life	n.a.	0.01 [0.01; 0.03]	n.a.	0.14 [0.07; 0.20]	0.06	[0.02; 0.20]	< 0.001
	3rd year of life	n.a.	0.01 [0.01; 0.03]	n.a.	0.17 [0.10; 0.25]	0.05	[0.02; 0.15]	< 0.001
	5th year of life	n.a.	0.02 [0.01; 0.04]	n.a.	0.19 [0.12; 0.27]	0.09	[0.04; 0.22]	< 0.001
	10th year of life	n.a.	0.09 [0.02; 0.27]	n.a.	0.23 [0.15; 0.32]	0.33	[0.07; 1.64]	0.176

*CI* confidence interval, *n* number of participants evaluated, *n.a.* not available, *OR* odds ratio

^a^IQWiG’s own calculation of the mortality rate from data on survival probability which is given in King et al. [26]. Information on the method used to estimate survival probability was missing in King et al. [26]. No absolute numbers of deaths or survivals available

^b^IQWiG´s own calculation: approximately determined from the data on mortality rates in the groups and self-estimated number *n*

^c^Parents of 40 of the 435 newborns did not attend the initial consultation. Therefore, 395 newborns were included in the intervention programme

The analyses of children up to the age of 5 showed consistent effects. The data showed a mortality rate of 0.01% at 2 and 3 years in the intervention group; the corresponding rate was 0.14% (at 2 years) and 0.17% (at 3 years) in the control group; the ORs were 0.06 (95% CI [0.02; 0.20]) at 2 years and 0.05 (95% CI [0.02; 0.15]) at 3 years, with *p* < 0.001 in both cases. The results at 5 years also showed a very large effect, with a mortality rate of 0.02% versus 0.19%; the OR was 0.09 (95% CI [0.04; 0.22]) with *p* < 0.001. The results for mortality at 10 years were not statistically significant (0.09% versus 0.23%; OR 0.33, 95% CI [0.07; 1.64], *p* = 0.176). As the estimated effect of the intervention was very large and consistent over several years of observation, no further sensitivity analyses were needed to ensure its robustness.

No suitable data were available for subgroup analyses.

As the mortality in children decreased by a factor of about 10 in the first 5 years of life, there was a very large difference between the intervention and the control group, which is unlikely to be explained by bias alone.

## Discussion

The results of this systematic review are based on a single, retrospective, historically controlled study evaluating a screening programme for SCD in Jamaica (King et al. [26]). It shows a decrease in mortality by a factor of 10 through screening for SCD followed by preventive measures. In our opinion, this large reduction in mortality may be explained only in part by bias associated with the comparison of non-concurrent groups in different decades or potential confounding factors, such as medical and technological progress. We therefore concluded that, in comparison with no screening, the data provide a hint of a benefit in favour of newborn screening for SCD. The sample size appears sufficient to draw this conclusion, as the precision of estimates is acceptable.

Consideration of linked evidence in addition to direct evidence would not have added much information to this review question. At best, i.e. if supporting evidence existed, the certainty of conclusions would still have remained relatively weak, as linked evidence studies cannot increase the reliability of direct evidence studies. If conflicting results from linked evidence existed, the direct evidence would still in most cases be regarded as more convincing, as linking evidence increases uncertainty [23].

The test procedure used in King et al.’s study [26] no longer complies with the latest laboratory standards. Established diagnostic test procedures for SCD are currently available: The results of test-positive newborns screened using tandem mass spectrometry or HPLC, for example, showed that they were actually affected by SCD, since no false-positive results were reported in selected studies (e.g. [29, 30]). Since the factors influencing test accuracy may vary, essential factors for the widespread use of such screening programmes are the training of laboratory personnel, laboratory standards, and the minimum amount of blood per sample to be analysed. The Jamaican screening programme includes prevention of infections and education of parents. These measures (e.g. penicillin prophylaxis) are also recommended as essential components of the early management of newborns with SCD in clinical guidelines in Western industrial countries [6, 13, 14, 31, 32], although in some countries, e.g. Germany, penicillin is not approved as a preventive measure for asymptomatic children with SCD. In these countries, as well as in Jamaica, the treatment of acute complications—currently and at the time of the study—includes immediate antibiotic treatment for fever and/or an enlarged spleen and blood transfusions for low Hb levels. It can therefore be assumed that the findings of King et al. [26] can be applied both to German and other healthcare systems.

This review excluded screening studies comparing a cohort of children screened and treated after birth with a cohort of children treated as soon as symptoms appeared [8, 33, 34]. This is because it can be assumed that in the latter cohort, children may be missed who died of SCD without being diagnosed; robust conclusions on SCD-related mortality cannot therefore be drawn. However, the data reported in these studies are in line with the decrease in mortality reported in King et al. [26], although the differences between the intervention and control groups are less pronounced. This is plausible, as the potential undetected SCD-related early deaths in the control groups may lead to an underestimation of the survival benefit in children with SCD detected by screening versus children with SCD identified after showing clinical symptoms.

As early as 1986, an RCT [12] showed that penicillin prophylaxis in asymptomatic children with SCD can reduce mortality by reducing the rate of infections. This also supports our conclusion that newborn screening for SCD lowers mortality. Against this background, the conduct of future RCTs to evaluate the benefits of newborn screening for SCD seems unethical and unlikely.

In April 2017, a consensus meeting of European experts (EuroBloodNet) was held, which discussed the organisation and methods of screening for SCD and made recommendations for implementation [35]. For example, the experts discussed which laboratory methods are appropriate (HPLC, capillary electrophoresis, isoelectric focussing, and tandem mass spectrometry) or what the recommended procedure should be after a positive screening result (e.g. confirming the positive screening result with another method using a second sample or re-testing with the same method) [35]. They also noted a great variety of screening and management strategies across European countries and that data on affected newborns were lacking for some regions. On the one hand, future research should therefore focus on regional SCD prevalence rates. On the other, screening and managing strategies should be evaluated to gain information on which strategies are the most suitable for the local healthcare systems and demographics.

### Recommendations in the literature

Our literature search identified 4 systematic reviews on newborn screening for SCD: one Cochrane review published in 2000 [36], 2 from the Galician Agency for Health Technology Assessment (original report from 2004 and update from 2013) [37, 38], and one from the Canadian Institute of Health Economics published in 2006 [32]. No review identified high-level evidence (i.e. RCTs) evaluating newborn screening for SCD but also included the study King et al. [26]. All note that no robust evidence to support newborn screening for SCD is available, but nevertheless mostly recommend this intervention.

### Limitations

Our results are based on a single, retrospective, historically controlled study in Jamaica with a high risk of bias (King et al. [26]). We therefore concluded that, in comparison with no screening, the data provide only a hint (i.e. the weakest certainty of conclusions according to IQWiG’s grading system) of a benefit in favour of newborn screening for SCD.

The control group in King et al.’s study [26] was also identified by newborn screening for SCD. Since no further specific preventive measures followed after diagnosis, the natural course of disease was investigated and this cohort was considered to be a suitable control group for the present review. Furthermore, only the test-positive newborns were followed up, which was plausible from an economical and practical perspective, even though the follow-up of all tested newborns would have resulted in a more precise data basis.

### Conclusion

The decrease in mortality by a factor of 10 is unlikely to be explained alone by the high risk of bias in the single retrospective study considered in our systematic review (largely due to the inclusion of historical controls). Therefore, in terms of mortality, the data suggest a benefit of newborn screening for SCD (followed by preventive measures) versus no newborn screening for SCD (weak certainty of conclusions).

## Supplementary Information

**Additional file 1.** PRISMA checklist.

**Additional file 2.** Search strategy.

**Additional file 3.** Publications lists.

## Data Availability

The full (German-language) report and protocol (Commission No. S18-01) are available on the Institute’s website (www.iqwig.de). The datasets supporting the conclusions of this article are included within the article (and its additional files).

## Abbreviations

CI: Confidence interval
Hb: Haemoglobin
HbA: Haemoglobin A
HbC: Haemoglobin C
HbS: Sickle cell haemoglobin
HTA: Health technology assessment
IQWiG: Institute for Quality and Efficiency in Health Care
OR: Odds ratio
PRISMA: Preferred Reporting Items for Systematic reviews and Meta-Analyses
RCT: Randomised controlled trial
SCD: Sickle cell disease
SCD-S/beta^0^: Heterozygous sickle cell disease (genotype combination HbS mutation and beta- thalassaemia mutation)
SCD-S/C: Heterozygous sickle cell disease (genotype combination HbS mutation and HbC mutation)
SCD-S/S: Homozygous sickle cell disease
