# Supplementary material for: Screening for sickle cell disease in newborns: a systematic review
Source: Syst Rev. 2020 Oct 30;9:250. doi: 10.1186/s13643-020-01504-5 (PMC7602310; doi:10.1186/s13643-020-01504-5)
Supplement: Supplementary file 1 — Additional file 1. PRISMA checklist. [file 13643_2020_1504_MOESM1_ESM.docx]

**Additional file 2:**

**Search strategy for direct evidence**

**1. MEDLINE**

***Search interface: Ovid***

- Ovid MEDLINE(R) 1946 to June Week 4 2020
- Ovid MEDLINE(R) In-Process & Other Non-Indexed Citations 1946 to July 06, 2020
- Ovid MEDLINE(R) Daily Update July 06, 2020
- Ovid MEDLINE(R) Epub Ahead of Print July 06, 2020

| # | Searches |
| --- | --- |
| 1 | exp Anemia, Sickle Cell/ |
| 2 | Hemoglobinopathies/ |
| 3 | (sickle cell* adj3 (disease* or anemia* or anaemia*)).ti,ab. |
| 4 | (hemoglobinopath* or haemoglobinopath*).ti,ab. |
| 5 | ((hemoglobin* or haemoglobin*) adj1 SC*).ti,ab. |
| 6 | or/1-5 |
| 7 | exp Infant/ |
| 8 | (newborn* or neonat* or pediatric* or infant*).ti,ab. |
| 9 | or/7-8 |
| 10 | Neonatal Screening/ |
| 11 | and/6,10 |
| 12 | *Mass Screening/ |
| 13 | screen*.ti,ab. |
| 14 | or/12-13 |
| 15 | and/6,9,14 |
| 16 | or/11,15 |
| 17 | 16 not (comment or editorial).pt. |
| 18 | 17 not (exp animals/ not humans.sh.) |

**2. PubMed**

***Search interface: NLM***

- PubMed - as supplied by publisher
- PubMed - in process
- PubMed – pubmednotmedline

| Search | Query |
| --- | --- |
| #1 | Search (sickle cell* [TIAB] AND (disease* [TIAB] OR anemia* [TIAB] OR anaemia* [TIAB])) |
| #2 | Search (hemoglobinopath* [TIAB] OR haemoglobinopath* [TIAB]) |
| #3 | Search ((hemoglobin* [TIAB] OR haemoglobin* [TIAB]) AND SC*[TIAB]) |
| #4 | Search (#1 OR #2 OR #3) |
| #5 | Search (newborn* [TIAB] OR neonat* [TIAB] OR pediatric* [TIAB] OR infant* [TIAB]) |
| #6 | Search screen*[TIAB] |
| #7 | Search (#4 AND #5 AND #6) |
| #8 | Search (#7 NOT medline[SB]) |

**3. EMBASE**

***Search interface: Ovid***

- Embase 1974 to 2020 July 06

| # | Searches |
| --- | --- |
| 1 | (sickle cell* adj3 (disease* or anemia* or anaemia*)).ti,ab. |
| 2 | (hemoglobinopath* or haemoglobinopath*).ti,ab. |
| 3 | ((hemoglobin* or haemoglobin*) adj1 SC*).ti,ab. |
| 4 | or/1-3 |
| 5 | exp infant/ |
| 6 | (newborn* or neonat* or pediatric* or infant*).ti,ab. |
| 7 | or/5-6 |
| 8 | newborn screening/ |
| 9 | and/4,8 |
| 10 | screen*.ti,ab. |
| 11 | and/4,7,10 |
| 12 | or/9,11 |
| 13 | 12 not medline.cr. |
| 14 | 13 not (exp animal/ not exp humans/) |
| 15 | 14 not (Conference Abstract or Conference Review or Editorial).pt. |

**4. The Cochrane Library**

***Search interface: Wiley***

- Cochrane Database of Systematic Reviews: Issue 7 of 12, July 2020
- Cochrane Central Register of Controlled Trials: Issue 7 of 12, July 2020

| ID | Search |
| --- | --- |
| #1 | [mh "Anemia, Sickle Cell"] |
| #2 | [mh ^Hemoglobinopathies] |
| #3 | (sickle cell* near/3 (disease* or anemia* or aenemia*)):ti,ab |
| #4 | (hemoglobinopath* or haemoglobinopath*):ti,ab |
| #5 | ((hemoglobin* or haemoglobin*) near/1 SC*):ti,ab |
| #6 | #1 OR #2 OR #3 OR #4 OR #5 |
| #7 | [mh Infant] |
| #8 | (newborn* or neonat* or pediatric* or infant*):ti,ab |
| #9 | #7 OR #8 |
| #10 | [mh ^"Neonatal Screening"] |
| #11 | #6 AND #10 |
| #12 | [mh ^"Mass Screening" [mj]] |
| #13 | screen*:ti,ab |
| #14 | #12 OR #13 |
| #15 | #6 AND #9 AND #14 |
| #16 | #11 OR #15 in Cochrane Reviews, Cochrane Protocols, Trials |

5. Health Technology Assessment Database

Search interface: Centre for Reviews and Dissemination

| Line | Search |
| --- | --- |
| 1 | (MeSH DESCRIPTOR Anemia, Sickle Cell EXPLODE ALL TREES) |
| 2 | (MeSH DESCRIPTOR Hemoglobinopathies) |
| 3 | (sickle cell* AND (disease* or anemia* or anaemia*)) |
| 4 | (hemoglobinopath* OR haemoglobinopath*) |
| 5 | ((hemoglobin* OR haemoglobin*) AND SC*) |
| 6 | (#1 OR #2 OR #3 OR #4 OR #5) |
| 7 | (MeSH DESCRIPTOR infant EXPLODE ALL TREES) |
| 8 | (newborn* or neonat* or pediatric* or infant*) |
| 9 | (#7 OR #8) |
| 10 | (MeSH DESCRIPTOR neonatal screening) |
| 11 | (#6 AND #10) |
| 12 | (MeSH DESCRIPTOR Mass Screening) |
| 13 | (screen*) |
| 14 | #12 OR #13 |
| 15 | #6 AND #9 AND #14 |
| 16 | #11 OR #15 |
| 17 | (#16) IN HTA |

**Search strategy in trial registries**

1. ClinicalTrials.gov

Provider: U.S. National Institutes of Health

- URL: <http://www.clinicaltrials.gov>
- Search interface: Basic Search

| Search strategy |
| --- |
| (sickle cell OR hemoglobinopathy OR hemoglobin sc disease) AND (screening OR chromatography OR hplc OR electrophoresis OR isoelectric focusing OR mass spectrometry) |

2.EU Clinical Trials Register

Provider: European Medicines Agency

- URL: <https://www.clinicaltrialsregister.eu/ctr-search/search>
- Search interface: Basic Search

| Search strategy |
| --- |
| ("sickle cell" OR hemoglobinopath* OR haemoglobinopath* OR "hemoglobin sc" OR "haemoglobin sc") AND ( screening OR chromatography OR hplc OR electrophoresis OR "isoelectric focusing" OR "mass spectrometry") |

3. International Clinical Trials Registry Platform Search Portal

Provider: World Health Organization

- URL: <http://apps.who.int/trialsearch/>
- Search interface: standard search

| Search strategy |
| --- |
| sickle cell AND screening OR sickle cell AND chromatography OR sickle cell AND hplc OR sickle cell AND electrophoresis OR sickle cell AND isoelectric focusing OR sickle cell AND mass spectrometry |
| hemoglobin* AND screening OR hemoglobin* AND chromatography OR hemoglobin* AND hplc OR hemoglobin* AND electrophoresis OR hemoglobin* AND isoelectric focusing OR hemoglobin* AND mass spectrometry |
| haemoglobin* AND screening OR haemoglobin* AND chromatography OR haemoglobin* AND hplc OR haemoglobin* AND electrophoresis OR haemoglobin* AND isoelectric focusing OR haemoglobin* AND mass spectrometry |
