# Supplementary material for: Screening for sickle cell disease in newborns: a systematic review
Source: Syst Rev. 2020 Oct 30;9:250. doi: 10.1186/s13643-020-01504-5 (PMC7602310; doi:10.1186/s13643-020-01504-5)
Supplement: Supplementary file 2 — Additional file 2. Search strategy. [file 13643_2020_1504_MOESM2_ESM.docx]

List of excluded publications (full text) with reasons for exclusion

Wrong population

1. Hassell KL. Population estimates of sickle cell disease in the U.S. Am J Prev Med 2010; 38(4 Suppl): S512-S521.

2. Jain D, Arjunan A, Sarathi V, Jain H, Bhandarwar A, Vuga M et al. Clinical events in a large prospective cohort of children with sickle cell disease in Nagpur, India: evidence against a milder clinical phenotype in India. Pediatr Blood Cancer 2016; 63(10): 1814-1821.

3. Lutcher CL, Huisman TH, Dorsey WM, Mayson S, Ludvigsen B, Smith AT. The role of a sickle cell center in comprehensive screening and counseling for sickle cell and related disorders. South Med J 1974; 67(3): 259-264.

4. Meloni T, Gallisai D, Dore A, Forteleoni G, Mela G. Neonatal screening for hemoglobinopathy in North Sardinia. Eur J Pediatr 1981; 137(2): 195-196.

5. Miller JM, Davis DC. Experience of a sickle cell screening program in Baltimore. J Natl Med Assoc 1979; 71(9): 839-841.

6. Paulukonis ST, Harris WT, Coates TD, Neumayr L, Treadwell M, Vichinsky E et al. Population based surveillance in sickle cell disease: methods, findings and implications from the California registry and surveillance system in hemoglobinopathies project (RuSH). Pediatr Blood Cancer 2014; 61(12): 2271-2276.

Wrong study intervention

1. Arduini GAO, Rodrigues LP, Trovo de Marqui AB. Mortality by sickle cell disease in Brazil. Rev Bras Hematol Hemoter 2017; 39(1): 52-56.

2. Grannum D, Lashley PM. The morbidity pattern of children with sickle cell disorders admitted to the Queen Elizabeth Hospital, Barbados (2009-2013). Trop Doct 2018; 48(1): 11-16.

3. Grosse SD, Odame I, Atrash HK, Amendah DD, Piel FB, Williams TN. Sickle cell disease in Africa: a neglected cause of early childhood mortality. Am J Prev Med 2011; 41(6 Suppl 4): S398-S405.

4. Hardie R, King L, Fraser R, Reid M. Prevalence of pneumococcal polysaccharide vaccine administration and incidence of invasive pneumococcal disease in children in Jamaica aged over 4 years with sickle cell disease diagnosed by newborn screening. Ann Trop Paediatr 2009; 29(3): 197-202.

5. Hayeems RZ, Bytautas JP, Miller FA. A systematic review of the effects of disclosing carrier results generated through newborn screening. J Genet Couns 2008; 17(6): 538-549.

6. Kavanagh PL, Sprinz PG, Vinci SR, Bauchner H, Wang CJ. Management of children with sickle cell disease: a comprehensive review of the literature. Pediatrics 2011; 128(6): e1552-e1574.

7. Leao LL, Aguiar MJ. Newborn screening: what pediatricians should know. J Pediatr (Rio J) 2008; 84(4 Suppl): S80-S90.

8. Lee A, Thomas P, Cupidore L, Serjeant B, Serjeant G. Improved survival in homozygous sickle cell disease: lessons from a cohort study. BMJ 1995; 311: 1600.

9. Lieberman L, Kirby M, Ozolins L, Mosko J, Friedman J. Initial presentation of unscreened children with sickle cell disease: the Toronto experience. Pediatr Blood Cancer 2009; 53(3): 397-400.

10. Macharia AW, Mochamah G, Uyoga S, Ndila CM, Nyutu G, Makale J et al. The clinical epidemiology of sickle cell anemia in Africa. Am J Hematol 2018; 93(3): 363-370.

11. Milne RI. Assessment of care of children with sickle cell disease: implications for neonatal screening programmes. BMJ 1990; 300(6721): 371-374.

12. O'Leary JD, Odame I, Pehora C, Chakraborty P, Crawford MW. Effectiveness of preoperative screening for sickle cell disease in a population with a newborn screening program: a cohort study. Can J Anaesth 2013; 60(1): 54-59.

13. Serjeant GR, Chin N, Asnani MR, Serjeant BE, Mason KP, Hambleton IR et al. Causes of death and early life determinants of survival in homozygous sickle cell disease: the Jamaican cohort study from birth. PLoS One 2018; 13(3): e0192710.

14. Streetly A, Grant C, Bickler G, Eldridge P, Bird S, Griffiths W. Variation in coverage by ethnic group of neonatal (Guthrie) screening programme in south London. BMJ 1994; 309(6951): 372-374.

15. Suijker MH, Roovers EA, Fijnvandraat CJ, Dors N, Rodrigues Pereira R, Giordano PC et al. Haemoglobinopathy in the 21st century: incidence, diagnosis and heel prick screening [Niederländisch]. Ned Tijdschr Geneeskd 2014; 158(33): a7365.

16. Wastnedge E, Waters D, Patel S, Morrison K, Goh MY, Adeloye D et al. The global burden of sickle cell disease in children under five years of age: a systematic review and meta-analysis. J Glob Health 2018; 8(2): 021103.

17. Wong HB, Kham S. Screening for haemoglobinopathies/thalassaemia utilising haematologic indices. J Singapore Paediatr Soc 1984; 26(3-4): 170-175.

18. Yawn BP, Buchanan GR, Afenyi-Annan AN, Ballas SK, Hassell KL, James AH et al. Management of sickle cell disease: summary of the 2014 evidence-based report by expert panel members. JAMA 2014; 312(10): 1033-1048.

19. Zanette AM, Goncalves Mde S, Bahia RC, Nogueira LV, Arruda SM. Sickle cell anemia: delayed diagnosis in Bahia, Brazil: a largely Afro-descendant population. Ethn Dis 2011; 21(2): 243-247.

Wrong control intervention

1. Update: newborn screening for sickle cell disease: California, Illinois, and New York, 1998. JAMA 2000; 284(11): 1373-1374.

2. Bardakdjian-Michau J, Bahuau M, Hurtrel D, Godart C, Riou J, Mathis M et al. Neonatal screening for sickle cell disease in France. J Clin Pathol 2009; 62(1): 31-33.

3. Bardakdjian-Michau J, Guilloud-Batailie M, Maier-Redelsperger M, Elion J, Girot R, Feingold J et al. Decreased morbidity in homozygous sickle cell disease detected at birth. Hemoglobin 2002; 26(3): 211-217.

4. Brown AK, Miller ST, Agatisa P. Care of infants with sickle cell disease: the ultimate objective of newborn screening; care of infants with sickle cell disease. Pediatrics 1989; 83(5): 897-900.

5. Centers for Disease Control and Prevention. Update: newborn screening for sickle cell disease; California, Illinois, and New York, 1998. MMWR Morb Mortal Wkly Rep 2000; 49(32): 729-731.

6. Diaz-Barrios V. New York's experience. Pediatrics 1989; 83(5): 872-875.

7. Ebomoyi W, Cherry FF. Prospective evaluation of targed filter paper screening for sickle cell disease: effectiveness and follow through. Int J Med Eng Inform 2010; 2(4): 376-388.

8. Eller R, Da Silva DB. Evaluation of a neonatal screening program for sickle-cell disease. J Pediatr (Rio J) 2016; 92(4): 409-413.

9. Galacteros F. Neonatal screening for sickle cell anemia in metropolitan France. For the group for neonatal screening of sickle cell anemia of the French Association for Screening and Prevention of Infant Handicaps (AFDPHE) [Französisch]. Pathol Biol (Paris) 1999; 47(1): 13-18.

10. Gaston M, Smith J, Gallagher D, Flournoy-Gill Z, West S, Bellevue R et al. Recruitment in the Cooperative Study of Sickle Cell Disease (CSSCD). Control Clin Trials 1987; 8(4 Suppl): 131S-140S.

11. Gill FM, Brown A, Gallagher D, Diamond S, Goins E, Grover R et al. Newborn experience in the cooperative study of sickle cell disease. Pediatrics 1989; 83(5): 827-829.

12. Githens JH, Lane PA, McCurdy RS, Houston ML, McKinna JD, Cole DM. Newborn screening for hemoglobinopathies in Colorado: the first 10 years. Am J Dis Child 1990; 144(4): 466-470.

13. Grover R. Program effects on decreasing morbidity and mortality: newborn screening in New York City. Pediatrics 1989; 83(5): 819-822.

14. Hayes RJ, Serjeant GR. Testing for the random occurrence of sickle cell disease in a study of 100,000 Jamaican newborns. J Trop Med Hyg 1990; 93(2): 127-132.

15. Jain D, Tokalwar R, Upadhye D, Colah R, Serjeant GR. Homozygous sickle cell disease in Central India & Jamaica: A comparison of newborn cohorts. Indian J Med Res 2020; 151(4): 326-332.

16. King LG, Bortolusso-Ali S, Cunningham-Myrie CA, Reid ME. Impact of a comprehensive sickle cell center on early childhood mortality in a developing country: the Jamaican experience. J Pediatr 2015; 167(3): 702-705.e701.

17. Law AS, Craven EM, Sarafidis EH. Screening of newborns for hemoglobinopathies: results in 5,484 patients. Del Med J 1985; 57(3): 161-164.

18. Le PQ, Ferster A, Cotton F, Vertongen F, Vermylen C, Vanderfaeillie A et al. Sickle cell disease from Africa to Belgium, from neonatal screening to clinical management. Med Trop (Mars) 2010; 70(5-6): 467-470.

19. Le PQ, Ferster A, Dedeken L, Vermylen C, Vanderfaeillie A, Rozen L et al. Neonatal screening improves sickle cell disease clinical outcome in Belgium. J Med Screen 2018; 25(2): 57-63.

20. Lerner NB, Platania BL, LaBella S. Newborn sickle cell screening in a region of Western New York State. J Pediatr 2009; 154(1): 121-125.

21. Lobel JS, Cameron BF, Johnson E, Smith D. The value of screening umbilical cord blood for hemoglobinopathy. Ohio State Med J 1984; 80(2): 140-142.

22. Lobel JS, Cameron BF, Johnson E, Smith D, Kalinyak K. Value of screening umbilical cord blood for hemoglobinopathy. Pediatrics 1989; 83(5): 823-826.

23. Lobo CL, Ballas SK, Domingos AC, Moura PG, Do Nascimento EM, Cardoso GP et al. Newborn screening program for hemoglobinopathies in Rio de Janeiro, Brazil. Pediatr Blood Cancer 2014; 61(1): 34-39.

24. Mack AK. Florida's experience with newborn screening. Pediatrics 1989; 83(5): 861-863.

25. Mason K, Gibson F, Gardner R, Warren L, Fisher C, Higgs D et al. Newborn screening for sickle cell disease: Jamaican experience. West Indian Med J 2015; 65(1): 18-26.

26. McGann PT, Ferris MG, Ramamurthy U, Santos B, De Oliveira V, Bernardino L et al. A prospective newborn screening and treatment program for sickle cell anemia in Luanda, Angola. Am J Hematol 2013; 88(12): 984-989.

27. Nussbaum RL, Powell C, Graham HL, Caskey CT, Fernbach DJ. Newborn screening for sickling hemoglobinopathies: Houston, 1976 to 1980. Am J Dis Child 1984; 138(1): 44-48.

28. Perrine RP, John P, Pembrey M, Perrine S. Sickle cell disease in Saudi Arabs in early childhood. Arch Dis Child 1981; 56(3): 187-192.

29. Quinn CT, Rogers ZR, Buchanan GR. Survival of children with sickle cell disease. Blood 2004; 103(11): 4023-4027.

30. Ralston KK, Kmetz DR, Keeling MM, Queenan JT. Screening for major hemoglobinopathies in newborn Blacks. J Ky Med Assoc 1981; 79(10): 649-651.

31. Sabarense AP, Lima GO, Silva LM, Viana MB. Survival of children with sickle cell disease in the comprehensive newborn screening programme in Minas Gerais, Brazil. Paediatr Int Child Health 2015; 35(4): 329-332.

32. Sabarense AP, Lima GO, Silva LM, Viana MB. Characterization of mortality in children with sickle cell disease diagnosed through the Newborn Screening Program. J Pediatr (Rio J) 2015; 91(3): 242-247.

33. Saint-Martin C, Romana M, Bibrac A, Brudey K, Tarer V, Divialle-Doumdo L et al. Universal newborn screening for haemoglobinopathies in Guadeloupe (French West Indies): a 27-year experience. J Med Screen 2013; 20(4): 177-182.

34. Serjeant GR, Serjeant BE. Management of sickle cell disease: lessons from the Jamaican cohort study. Blood Rev 1993; 7(3): 137-145.

35. Serjeant GR, Serjeant BE, Mason KP, Happich M, Kulozik AE. Beta-thalassemia mutations in Jamaica: geographic variation in small communities. Hemoglobin 2018; 42(5-6): 294-296.

36. Shafer FE, Lorey F, Cunningham GC, Klumpp C, Vichinsky E, Lubin B. Newborn screening for sickle cell disease: 4 years of experience from California's newborn screening program. J Pediatr Hematol Oncol 1996; 18(1): 36-41.

37. Soares LF, Rocha OA, De Oliveira EH, Vieira JF. Neonatal screening in the state of Piaui: an urgent need; a study on the prevalence of sickle cell disease in newborns. Rev Bras Hematol Hemoter 2012; 34(5): 392-393.

38. Sommet J, Alberti C, Couque N, Verlhac S, Haouari Z, Mohamed D et al. Clinical and haematological risk factors for cerebral macrovasculopathy in a sickle cell disease newborn cohort: a prospective study. Br J Haematol 2016; 172(6): 966-977.

39. Sprinz P, Lemke K, Padbury J, Farrow C. Newborn screening for hemoglobinopathies in Rhode Island, 2017. R I Med 2018; 101(7): 17-20.

40. Streetly A, Clarke M, Downing M, Farrar L, Foo Y, Hall K et al. Implementation of the newborn screening programme for sickle cell disease in England: results for 2003-2005. J Med Screen 2008; 15(1): 9-13.

41. Streetly A, Latinovic R, Hall K, Henthorn J. Implementation of universal newborn bloodspot screening for sickle cell disease and other clinically significant haemoglobinopathies in England: screening results for 2005-7. J Clin Pathol 2009; 62(1): 26-30.

42. Streetly A, Sisodia R, Dick M, Latinovic R, Hounsell K, Dormandy E. Evaluation of newborn sickle cell screening programme in England: 2010-2016. Arch Dis Child 2018; 103(7): 648-653.

43. Telfer P, Coen P, Chakravorty S, Wilkey O, Evans J, Newell H et al. Clinical outcomes in children with sickle cell disease living in England: a neonatal cohort in East London. Haematologica 2007; 92(7): 905-912.

44. Therrell BL Jr, Lloyd-Puryear MA, Eckman JR, Mann MY. Newborn screening for sickle cell diseases in the United States: a review of data spanning 2 decades. Semin Perinatol 2015; 39(3): 238-251.

45. Vichinsky E, Hurst D, Earles A, Kleman K, Lubin B. Newborn screening for sickle cell disease: effect on mortality. Pediatrics 1988; 81(6): 749-755.

46. Wang WC. Newborn hemoglobinopathy screening in Tennessee: current status. J Tenn Med Assoc 1989; 82: 473-476.

47. Wang Y, Liu G, Caggana M, Kennedy J, Zimmerman R, Oyeku SO et al. Mortality of New York children with sickle cell disease identified through newborn screening. Genet Med 2015; 17(6): 452-459.

48. West R, Ashcraft P, Becton D. Newborn screening for hemoglobinopathies in Arkansas: first two years' experience. J Ark Med Soc 1992; 88(8): 382-386.

Wrong outcomes

1. Gibbons C, Geoghegan R, Conroy H, Lippacott S, O'Brien D, Lynam P et al. Sickle cell disease: time for a targeted neonatal screening programme. Ir Med J 2015; 108(2): 43-45.

Wrong study type

1. Panel cites treatment success as impetus for hemoglobinopathy screening of newborns. Clin Pharm 1987; 6: 511.

2. Consensus development summaries: newborn screening for sickle cell disease and other hemoglobinophathies. Conn Med 1987; 51: 459-463.

3. Newborn screening for sickle cell disease and other hemoglobinopathies. JAMA 1987; 258(9): 1205-1209.

4. Screening checks newborns for galactosemia, hemoglobinopathy. J Okla State Med Assoc 1991; 84(3): 124-125.

5. Sickle cell disease: guideline overview. J Natl Med Assoc 1993; 85(8): 581-583.

6. Sickle cell disease: comprehensive screening and management in newborns and infants. Clin Pract Guidel Quick Ref Guide Clin 1993; (6): 1-13.

7. Mortality among children with sickle cell disease identified by newborn screening during 1990-1994: California, Illinois, and New York. JAMA 1998; 279(14): 1059-1060.

8. Correction: neonatal screening for sickle cell disease in France (J Clin Pathol 2008;62:31–3). J Clin Pathol 2009; 62(9): 864.

9. Abildgaard CF, Winston C. Newborn hemoglobinopathy screening. West J Med 1990; 153(6): 651.

10. Alexander-Reindorf C. Reduction of the infant mortality rate in the West Indies. J Natl Med Assoc 2008; 100(12): 1482.

11. Almeida AM, Henthorn JS, Davies SC. Neonatal screening for haemoglobinopathies: the results of a 10-year programme in an English Health Region. Br J Haematol 2001; 112(1): 32-35.

12. Amid A, Odame I. Improving outcomes in children with sickle cell disease: treatment considerations and strategies. Paediatr Drugs 2014; 16(4): 255-266.

13. Anglin S. Screen test for all. Nurs Stand 2006; 20(47): 28-29.

14. Anglin S. Sickle cell and thalassaemia screening: early care. Pract Midwife 2007; 10(9): 22-25.

15. Anionwu EN. Sickle cell disease: screening and counselling in the antenatal and neonatal period: part 2. Midwife Health Visit Community Nurse 1983; 19: 440-443.

16. Anionwu EN. Sickle cell disease: screening and counselling in the antenatal and neonatal period: part I. Midwife Health Visit Community Nurse 1983; 19: 402-406.

17. Armbruster DA. Neonatal hemoglobinopathy screening. Lab Med 1990; 21(12): 815-822.

18. Ballas SK, Park D, Shafer FE, Lorey F. Newborn screening for sickle cell disease. J Pediatr Hematol Oncol 1996; 18(4): 418.

19. Berg AO. Sickle cell disease: screening, diagnosis, management, and counseling in newborns and infants. J Am Board Fam Pract 1994; 7(2): 134-140.

20. Brosco JP, Grosse SD, Ross LF. Universal state newborn screening programs can reduce health disparities. JAMA Pediatrics 2015; 169(1): 7-8.

21. Brozovic M, Anionwu E. Sickle cell disease in Britain. J Clin Pathol 1984; 37(12): 1321-1326.

22. Buchanan GR. Sickle cell disease: Recent advances. Curr Probl Pediatr 1993; 23(6): 219-229.

23. Cassetta RA. Sickle cell guidelines stress screening. Am Nurse 1993; 25(6): 9.

24. Castilla-Rodriguez I, Cela E, Vallejo-Torres L, Valcarcel-Nazco C, Dulin E, Espada M et al. Cost-effectiveness analysis of newborn screening for sickle-cell disease in Spain. Expert Opin Orphan D 2016; 4(6): 567-575.

25. Cavazzana M, Stanislas A, Remus C, Duwez P, Renoult J, Cretet J et al. Evidence for the widespread use of neonatal screening for sickle cell disease [Französisch]. Med Sci (Paris) 2018; 34(4): 309-311.

26. Cela E, Bellon JM, De la Cruz M, Belendez C, Berrueco R, Ruiz A et al. National registry of hemoglobinopathies in Spain (REPHem). Pediatr Blood Cancer 2017; 64(7): e26322.

27. Centers for Disease Control and Prevention. Mortality among children with sickle cell disease identified by newborn screening during 1990-1994: California, Illinois, and New York. MMWR Morb Mortal Wkly Rep 1998; 47(9): 169-172.

28. Chapman CS. Neonatal screening for haemoglobinopathies. Clin Lab Haematol 1999; 21(4): 229-234.

29. Chaturvedi S, DeBaun MR. Evolution of sickle cell disease from a life-threatening disease of children to a chronic disease of adults: the last 40 years. Am J Hematol 2016; 91(1): 5-14.

30. Cope A, Darbyshire PJ. Sickle cell disease, update on management. Paediatr Child Health (Oxford) 2013; 23(11): 480-485. 480.

31. Davies SC, Cronin E, Gill M, Greengross P, Hickman M, Normand C. Screening for sickle cell disease and thalassaemia: a systematic review with supplementary research. Health Technol Assess 2000; 4(3): i-v, 1-99.

32. Davies SC, Oni L. Sickle cell disease screening programs: integration into managed care. Dis Manag Health Out 2001; 9(6): 295-304.

33. De Montalembert M. Management of children with sickle cell anemia: a collaborative work [Französisch]. Arch Pediatr 2002; 9(11): 1195-1201.

34. Earles A. Nursing perspective. Pediatrics 1989; 83(5): 901-902.

35. Ebomoyi EW. Neonatal screening for sickle cell disease, caveats about potential cure with innovative medical technology and the relevant evidence-based health education. Int J Med Eng Inform 2013; 5(1): 46-59.

36. Eckman JR, Kinney TR, Harris MS. Newborn screening for hemoglobinopathies: facilitation by a TASCS force. Ann N Y Acad Sci 1989; 565: 376-378.

37. Emodi I. In the absence of neonatal screening facilities for sickle cell anaemia. Ann Trop Paediatr 2000; 20(1): 77-78.

38. Epps RP. Perspective from the National Medical Association. Pediatrics 1989; 83(5): 911.

39. Erbe RW. Issues in newborn genetic screening. Birth Defects Orig Artic Ser 1981; 17(1): 167-179.

40. Erickson S, Smith JC. The Tennessee Newborn Hemoglobinopathy Screening Program. J Tenn Med Assoc 1987; 80(4): 223-224.

41. Francis YF. Screening and genetic counseling programs for sickle cell trait and sickle cell anemia. J Am Med Wom Assoc 1974; 29(9): 406-410.

42. Frempong T, Pearson HA. Newborn screening coupled with comprehensive follow-up reduced early mortality of sickle cell disease in Connecticut. Conn Med 2007; 71(1): 9-12.

43. Garrick MD, Dembure P, Guthrie R. Sickle-cell anemia and other hemoglobinopathies: procedures and strategy for screening employing spots of blood on filter paper as specimens. N Engl J Med 1973; 288(24): 1265-1268.

44. Gessner BD, Teutsch SM, Shaffer PA. A cost-effectiveness evaluation of newborn hemoglobinopathy screening from the perspective of state health care systems. Early Hum Dev 1996; 45(3): 257-275.

45. Gima AS. Sickle cell screening: considerations in approaching the population at risk. J Natl Med Assoc 1975; 67(6): 450-454.

46. Giordano P, Poland D, Harteveld K. Neonatal screening for hemoglobinopathy [Niederländisch]. Huisarts Wet 2011; 54(6): 343.

47. Giorgio AJ, Boggs DR. Large scale screening for hemoglobinopathies, utilizing electrophoresis. Am J Public Health 1974; 64(10): 993-995.

48. Githens JH. Sickle cell screening. Rocky Mt Med J 1979; 76(1): 21-25.

49. Glader BE. Screening for anemia and erythrocyte disorders in children. Pediatrics 1986; 78(2): 368-369.

50. Goldman DW. Screening newborns for hemoglobinopathies. JAMA 1988; 259(2): 219.

51. Gross T. Newborn screening for sickle cell disease. Pediatrics 1989; 83(4): 629-631.

52. Grossman LK. On newborn sickle cell screening in NYC. Am J Public Health 1983; 73(10): 1216-1217.

53. Guthrie R. Techniques and efficacy of screening: newborn screening. Pediatrics 1989; 83(5): 836-838.

54. Harris MS, Eckman JR. Approaches to screening: Georgia's experience with newborn screening; 1981 to 1985. Pediatrics 1989; 83(5): 858-860.

55. Henry DD. Public presentations: parental perspective. Pediatrics 1989; 83(5): 910.

56. Henthorn JS, Almeida AM, Davies SC. Neonatal screening for sickle cell disorders. Br J Haematol 2004; 124(3): 259-263.

57. Hernandez S. Social work perspective. Pediatrics 1989; 83(5): 903-905.

58. Holtzman NA. Perspective from the American Academy of Pediatrics. Pediatrics 1989; 83(5): 913-914.

59. Holtzman NA, Leonard CO, Farfel MR. Issues in antenatal and neonatal screening and surveillance for hereditary and congenital disorders. Annu Rev Public Health 1981; 2: 219-251.

60. Hurst D. Northern California's experience. Pediatrics 1989; 83(5): 868-871.

61. Jinks DC, Vanderford M, Fielding Hejtmancik J, McCabe ERB. Molecular genetic approach to newborn screening for sickle cell disease. Ann N Y Acad Sci 1989; 565(1): 434.

62. Kaback MM. Population screening for genetic disorders in California. UCLA Forum Med Sci 1978; 20: 207-219.

63. Karnon J, Zeuner D, Ades AE, Efimba W, Brown J, Yardumian A. The effects of neonatal screening for sickle cell disorders on lifetime treatment costs and early deaths avoided: a modelling approach. J Public Health Med 2000; 22(4): 500-511.

64. Kinney TR, Sawtschenko M, Whorton M, Shearin J, Stine C, Hofman L et al. Techniques' comparison and report of the North Carolina experience. Pediatrics 1989; 83(5): 843-848.

65. Kmietowicz Z. Screening for sickle cell disease and thalassaemia saving lives. BMJ 2004; 329: 69.

66. Kolata G. Panel urges newborn sickle cell screening. Science 1987; 236(4799): 259-260.

67. Kuznik A, Habib AG, Munube D, Lamorde M. Newborn screening and prophylactic interventions for sickle cell disease in 47 countries in sub-Saharan Africa: a cost-effectiveness analysis. BMC Health Serv Res 2016; 16: 304.

68. Lane PA, Eckman JR, Tsevat J, Wong JB, Pauker SG, Steinberg MH. Cost-effectiveness of neonatal screening for sickle cell disease: editorial correspondence. J Pediatr 1992; 120(1): 162-163.

69. Leary WE. Screening of all newborns urged for sickle-cell disease. New York Times 1993: c11.

70. Lee E. Sickle cell anemia. Office and Emergency Pediatrics 2000; 13(1): 14-20.

71. Lima ARG, Ribeiro VS, Nicolau DI. Trends in mortality and hospital admissions of sickle cell disease patients before and after the newborn screening program in Maranhao, Brazil. Rev Bras Hematol Hemoter 2015; 37(1): 12-16.

72. Lin K, Barton MB. Screening for hemoglobinopathies in newborns: reaffirmation update for the U.S. Preventive Services Task Force [online]. 09.2007 [Zugriff: 14.02.2019]. URL: https://www.ahrq.gov/downloads/pub/prevent/pdfser/Sicklecelles.pdf.

73. Lin KW. Screening for sickle cell disease in newborns. Am Fam Physician 2009; 79(6): 507-508.

74. Long SS. High cost effectiveness of newborn screening for sickle cell disease in resource-limited Angola. J Pediatr 2015; 167(6): 1179-1182.

75. Makani J, Soka D, Rwezaula S, Krag M, Mghamba J, Ramaiya K et al. Health policy for sickle cell disease in Africa: experience from Tanzania on interventions to reduce under-five mortality. Trop Med Int Health 2015; 20(2): 184-187.

76. Manu Pereira M, Corrons JL. Neonatal haemoglobinopathy screening in Spain. J Clin Pathol 2009; 62(1): 22-25.

77. McGann PT. Improving survival for children with sickle cell disease: newborn screening is only the first step. Paediatr Int Child Health 2015; 35(4): 285-286.

78. McGann PT, Grosse SD, Santos B, De Oliveira V, Bernardino L, Kassebaum NJ et al. A cost-effectiveness analysis of a pilot neonatal screening program for sickle cell anemia in the Republic of Angola. J Pediatr 2015; 167(6): 1314-1319.

79. McGann PT, Nero AC, Ware RE. Current management of sickle cell anemia. Cold Spring Harb Perspect Med 2013; 3(8): a011817.

80. Meschino WS, Gibbons CA, Allanson J, Blaine SM, Cremin C, Dorman H et al. Genetics: newborn screening for sickle cell anemia. Can Fam Physician 2009; 55(10): 1001.

81. Moreira RM, Estevao Ida F, Melo DG. Critical analysis of the neonatal screening program for hemoglobinopathies. Rev Bras Hematol Hemoter 2011; 33(4): 318-320.

82. Motulsky AG. Screening for sickle cell hemoglobinopathy and thalassemia. Isr J Med Sci 1973; 9(9): 1341-1349.

83. National Institutes of Health. Newborn screening for sickle cell disease and other hemoglobinopathies. Natl Inst Health Consens Dev Conf Consens Statement 1987; 6(9): 1-22.

84. Natowicz M. Newborn screening: setting evidence-based policy for protection. N Engl J Med 2005; 353(9): 867-870.

85. Naylor EW. Recent developments in neonatal screening. Semin Perinatol 1985; 9(3): 232-249.

86. Noguera NI, Bragos IM, Morisoli L, Milani AC. Screening for hemoglobinopathies in neonates in Argentina. Haematologica 1999; 84(5): 468-470.

87. North AF. Screening in child health care: where are we now and where are we going? Pediatrics 1974; 54(5): 631-640.

88. O'Brien RT. Perspectives in sickle cell disease screening. South Med J 1974; 67(11): 1269-1271.

89. Ohene-Frempong K. Selected testing of newborns for sickle cell disease. Pediatrics 1989; 83(5): 879-880.

90. Oni L. A sickle cell milestone. Br J Nurs 2009; 18(14): 842.

91. Pai GS, Houser PM. Neonatal screening for sickling hemoglobinopathies in South Carolina: can the promise be fulfilled? J S C Med Assoc 1987; 83: 243-245.

92. Panepinto JA, Magid D, Rewers MJ, Lane PA. Universal versus targeted screening of infants for sickle cell disease: a cost-effectiveness analysis. J Pediatr 2000; 136(2): 201-208.

93. Parkhurst J. Newborn metabolic disorder screening program: sickle cell disease and other hemoglobinopathies. J Okla State Med Assoc 1997; 90(6): 256-257.

94. Patel J, Serjeant GR. Newborn screening for sickle cell disease in India: the need for defining optimal clinical care. Indian J Pediatr 2014; 81(3): 229-230.

95. Pearson HA. A neonatal program for sickle cell anemia. Adv Pediatr 1986; 33: 381-400.

96. Pearson HA, O'Brien RT. Sickle cell testing programs. J Pediatr 1972; 81(6): 1201-1204.

97. Pearson HA, O'Brien RT. Sickle cell screening in newborns. Am J Dis Child 1976; 130(8): 799.

98. Pearson HA, O'Brien RT, McIntosh S, Aspnes GT, Yang MM. Routine screening of umbilical cord blood for sickle cell diseases. JAMA 1974; 227(4): 420-421.

99. Pegelow CH, Pitel P, Judisch J, Randall-David E, Siderits P, Ausbon W. Screening newborn infants for sickle hemoglobin. J Fla Med Assoc 1988; 75(10): 670-675.

100. Peters C, Miller J, Abel SL, McMillan SK, Getchell JP, Giller RH et al. Iowa newborn hemoglobinopathy screening and comprehensive care: a model for rural states. J Pediatr Hematol Oncol 1996; 18(4): 416-418.

101. Piety NZ, Shevkoplyas SS. Paper-based diagnostics: rethinking conventional sickle cell screening to improve access to high-quality health care in resource-limited settings. IEEE Pulse 2017; 8(3): 42-46.

102. Prabhakar H, Haywood C Jr, Molokie R. Sickle cell disease in the United States: looking back and forward at 100 years of progress in management and survival. Am J Hematol 2010; 85(5): 346-353.

103. Quinn CT. Sickle cell disease in childhood: from newborn screening through transition to adult medical care. Pediatr Clin North Am 2013; 60(6): 1363-1381.

104. Robitaille N, Delvin EE, Hume HA. Newborn screening for sickle cell disease: a 1988-2003 Quebec experience. Paediatr Child Health 2006; 11(4): 223-227.

105. Rowley PT. Newborn screening for sicle-cell disease. Benefits and burdens. N Y State J Med 1978; 78: 42-44.

106. Rowley PT. Newborn screening for hemoglobinopathies. Semin Perinatol 1990; 14(6): 483-487.

107. Rutkow IM, Lipton JM. Some negative aspects of state health departments' policies related to screening for sickle cell anemia. Am J Public Health 1974; 64: 217-221.

108. Schulte Strathaus R. Neugeborenen-Screening bei Sichelzellanämie gefordert. Med Monatsschr Pharm 2016; 39(4): 175.

109. Scott RB. Screening newborn infants for sickle cell disease: participation of comprehensive centers for sickle cell disease. Am J Pediatr Hematol Oncol 1988; 10(1): 3-4.

110. Scott RB. Survey of comprehensive centers for sickle cell disease. Pediatrics 1989; 83(5): 908-909.

111. Scott RB, Castro O. Screening for sickle cell hemoglobinopathies. JAMA 1979; 241(11): 1145-1147.

112. Scott RB, Harrison DL. Screening of the umbilical cord blood for sickle cell disease: utilization and implementation. Am J Pediatr Hematol Oncol 1982; 4(2): 202-205.

113. Serjeant BE, Forbes M, Williams LL, Serjeant GR. Screening cord bloods for detection of sickle cell disease in Jamaica. Clin Chem 1974; 20(6): 666-669.

114. Serjeant G, Serjeant B. Neonatal screening for sickle hemoglobin. Am J Clin Pathol 1979; 72(2): 251.

115. Serjeant GR. Screening for sickle-cell disease in Brazil. Lancet 2000; 356(9224): 168-169.

116. Shahidi NT. Newborn screening for sickle cell disease and other abnormal hemoglobins. Wis Med J 1988; 87(6): 21-22.

117. Shook LM, Ware RE. Sickle cell screening in Europe: the time has come. Br J Haematol 2018; 183(4): 534-535.

118. Smith JA, Kinney TR. Sickle cell disease: screening and management in newborns and infants. Am Fam Physician 1993; 48(1): 95-102.

119. Sprinkle RH, Hynes DM, Konrad TR. Is universal neonatal hemoglobinopathy screening cost-effective? Arch Pediatr Adolesc Med 1994; 148: 461-469.

120. Stuart J. Management of sickle-cell disease. J Clin Pathol Suppl (R Coll Pathol) 1974; 8: 26-31.

121. Tewari S, Rees D. Morbidity pattern of sickle cell disease in India: a single centre perspective. Indian J Med Res 2013; 138(3): 288-290.

122. Therrell BL Jr, Simmank JL, Wilborn M. Experiences with sickle hemoglobin testing in the Texas Newborn Screening Program. Pediatrics 1989; 83(5): 864-867.

123. Thomas R, Holbrook T. Sickle cell disease: ways to reduce morbidity and mortality. Postgrad Med 1987; 81(5): 265-8, 273-80.

124. Tsevat J, Wong JB, Pauker SG, Steinberg MH. Neonatal screening for sickle cell disease: a cost-effectiveness analysis. J Pediatr 1991; 118(4 Pt 1): 546-554.

125. Tubman VN, Field JJ. Sickle solubility test to screen for sickle cell trait: what's the harm? Hematology Am Soc Hematol Educ Program 2015; 2015(1): 433-435.

126. U.S. Preventive Services Task Force. Screening for sickle cell disease in newborns: recommendation statement. Am Fam Physician 2008; 77(9): 1300-1302.

127. U.S. Public Health Service. Newborn screenin. Am Fam Physician 1994; 50(2): 354-358.

128. Venable V. Should screening for sickle cell anemia by comprehensive? J Nurse Midwifery 1977; 22(3): 28-29.

129. Walker B Jr. Public health perspective on newborn screening for hemoglobinopathies. Pediatrics 1989; 83(5): 912.

130. Wang WC. Sickle cell disease in children. Clin Adv Hematol Oncol 2011; 9(7): 554-556.

131. Wang WC. Newborn screening for sickle cell disease: necessary but not sufficient. J Pediatr (Rio J) 2015; 91(3): 210-212.

132. Webb ZO. Sickle cell anemia: a clinical screening survey. J Natl Med Assoc 1972; 64(3): 197-199.

133. West R, Hale C. Overview of newborn screening in Arkansas. J Ark Med Soc 1995; 92(7): 329-333.

134. Wethers D, Pearson H, Gaston M. Newborn screening for sickle cell disease and other hemoglobinopathies. Pediatrics 1989; 83(5): 813-914.

135. Wethers DL. Sickle cell disease in childhood; part I: laboratory diagnosis, pathophysiology and health maintenance. Am Fam Physician 2000; 62(5): 1013-20, 1027-8.

136. Whitten CF. Perspective from the National Association for Sickle Cell Disease. Pediatrics 1989; 83(5): 906-907.

137. Wierenga KJ. Neonatal screening for sickle-cell disease [Niederländisch]. Ned Tijdschr Geneeskd 1997; 141(4): 184-187.

138. Wilson RE, Krishnamurti L, Kamat D. Management of sickle cell disease in primary care. Clin Pediatr (Phila) 2003; 42(9): 753-761.
